# Multivariate Statistical Analysis of Distribution of Deep-Water Gorgonian Corals in Relation to Seabed Topography on the Norwegian Margin

**DOI:** 10.1371/journal.pone.0043534

**Published:** 2012-08-17

**Authors:** Ruiju Tong, Autun Purser, Vikram Unnithan, Janine Guinan

**Affiliations:** 1 Jacobs University Bremen, Bremen, Germany; 2 INFOMAR, Marine and Geophysics Programme, Geological Survey of Ireland, Dublin, Ireland; Leibniz Center for Tropical Marine Ecology, Germany

## Abstract

Investigating the relationship between deep-water coral distribution and seabed topography is important for understanding the terrain habitat selection of these species and for the development of predictive habitat models. In this study, the distribution of the deep-water gorgonians, *Paragorgia arborea* and *Primnoa resedaeformis*, in relation to terrain variables at multiple scales of 30 m, 90 m and 170 m were investigated at Røst Reef, Traena Reef and Sotbakken Reef on the Norwegian margin, with Ecological Niche Factor Analysis applied. To date, there have been few published studies investigating this aspect of gorgonian distribution. A similar correlation between the distribution of *P. arborea* and *P. resedaeformis* and each particular terrain variable was found at each study site, but the strength of the correlation between each variable and distribution differed by reef. The terrain variables of bathymetric position index (BPI) and curvature at analysis scales of 90 m or 170 m were most strongly linked to the distribution of both species at the three geographically distinct study sites. Both gorgonian species tended to inhabit local topographic highs across all three sites, particularly at Sotbakken Reef and Traena Reef, with both species observed almost exclusively on such topographic highs. The tendency for observed *P. arborea* to inhabit ridge crests at Røst Reef was much greater than was indicated for *P. resedaeformis*. This investigation identifies the terrain variables which most closely correlate with distribution of these two gorgonian species, and analyzes their terrain habitat selection; further development of predictive habitat models may be considered essential for effective management of these species.

## Introduction

Deep-water gorgonian corals are found throughout the world’s oceans and Mediterranean Sea, with the majority reported at depths of 200–1,000 m [1]. *Paragorgia arborea* and *Primnoa resedaeformis* are amongst the largest deep-water gorgonians, with colonies reaching heights of 50–250 cm [2]. These species are commonly found in the North Atlantic [1], [3], [4], [5], often in water masses with temperatures of 6–8°C and high salinity [2]. Both species produce tree-like colony morphologies, which provide habitats within and between colonies, commonly utilized by numerous invertebrate species [3], [6], [7]. However, due to their arborescent morphology and assumed slow growth, these gorgonian corals are vulnerable to anthropogenic impacts such as bottom trawling and gillnet fishing, and are often reported as fishing bycatch [2], [8], [9], [10], [11].

Deep-water corals (DWCs) are typically observed in abundance in areas of pronounced topographic relief [1], [3], [5], [12], [13], [14], [15]. Seabed topographic features may control the coral distribution indirectly by governing current regimes, thereby influencing the flux of suspended food material [1], [12], [16], [17], [18], [19], and also by influencing sediment distribution that is important for initial coral settlement [20], [21]. For the past two decades multibeam echosounder surveys have been providing detailed bathymetry data suitable for broad characterization of large regions of the seafloor. Wilson et al. [21] comprehensively summarized the terrain variables (topography descriptors) derived from such multibeam bathymetry data into four types – 1) slope type, 2) aspect type, 3) curvature and bathymetric position index (BPI) type, and 4) terrain variability type. Characterization of the seabed topography in terms of such terrain variables at multiple scales may have ecological relevance to the distribution of benthic fauna, with these variables acting as proxies for bottom current velocity or substrate type at local and regional scales [18], [21].

Information on the distribution and population densities of deep-water gorgonians is required for conservation and management of these species, but such data is scarce given the high costs associated with sampling and surveying in deep sea. Predictive habitat suitability may be used as proxies for species distribution, or for optimizing mapping strategies [16], [17], [18], [22], [23], [24], [25], [26], i.e. to indicate seafloor areas which may contain corals and coral ecosystems with the potential to be put at risk by human activities such as bottom trawling, long-line or gillnet fishing [8], [27]. Gridded bathymetry data and the derived terrain variables are often adopted as descriptors of seabed topography when developing predictive habitat models. Investigating the relationship between the distribution of deep-water gorgonians and the associated terrain variables will improve our understanding of the terrain habitat requirements of the species, and is also important in developing such predictive models [12]. However, to date, investigations of how these terrain variables relate to DWC distribution have focused primarily on hard scleractinian corals rather than gorgonians [12], [17], [18], [24], [25].

**Figure 1:**
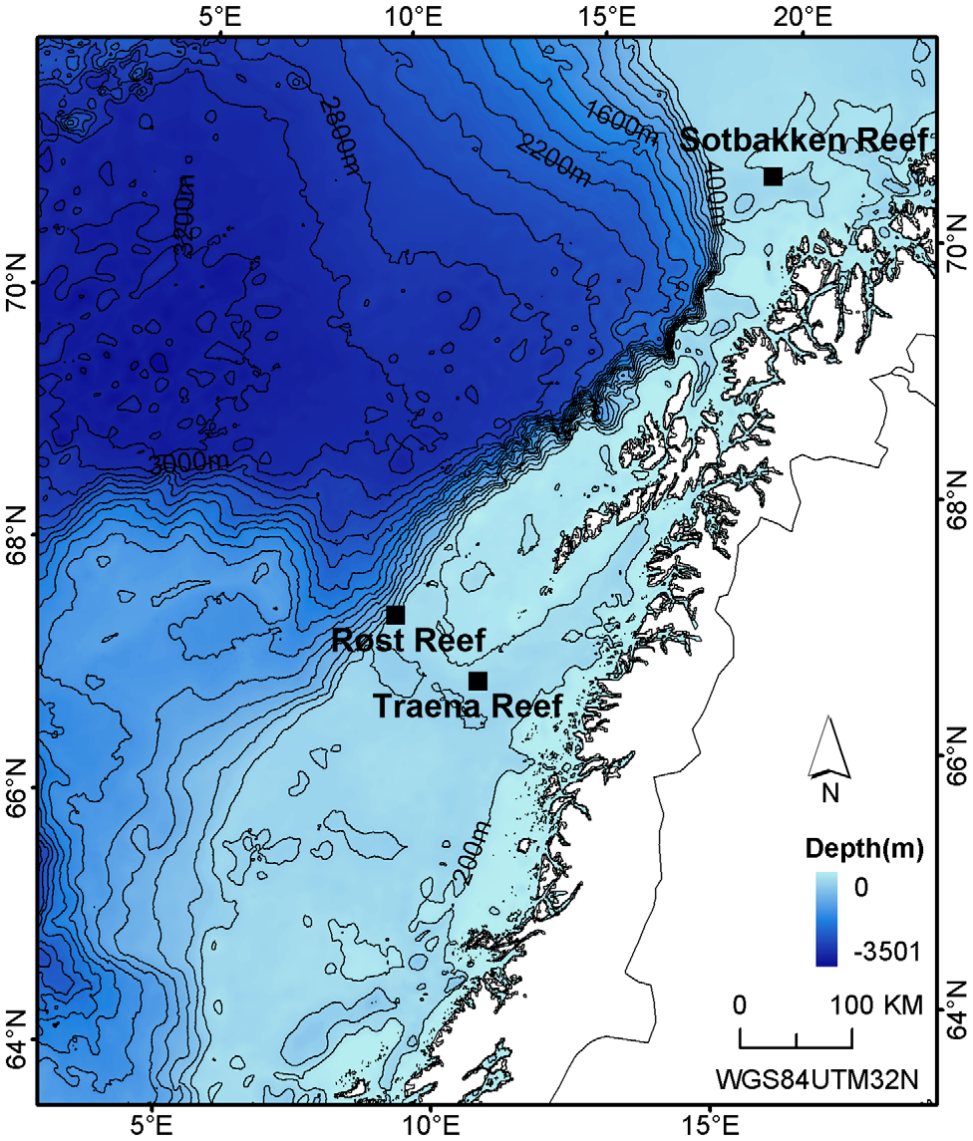
Three study sites on the Norwegian margin – Sotbakken Reef, Røst Reef and Traena Reef.

This study investigated the distribution of the deep-sea gorgonians, *P. arborea* and *P. resedaeformis*, in relation to seabed topography at Røst Reef and Traena Reef, and *P. arborea* at Sotbakken Reef on the Norwegian margin. These three reef complexes vary greatly in topography and environmental conditions, with Røst Reef located on the shelf margin, Sotbakken Reef at the northern tip of the Norwegian Shelf and Traena Reef on the inner shelf (Figure 1). This study aims to address the following questions: (1) which, if any, particular terrain variables correlate most closely with the observed distribution of *P. arborea* and *P. resedaeformis* at Røst Reef and Traena Reef, and of *P. arborea* at Sotbakken Reef; (2) what, if any, trends exist in habitat selection of *P. arborea* and *P. resedaeformis* at Røst Reef and Traena Reef, and of *P. arborea* at Sotbakken Reef.

## Materials and Methods

### Ethics Statement

The survey of the reefs presented in this study was permitted by the Institute of Marine Research, Norway.

### Study Areas

**Figure 2:**
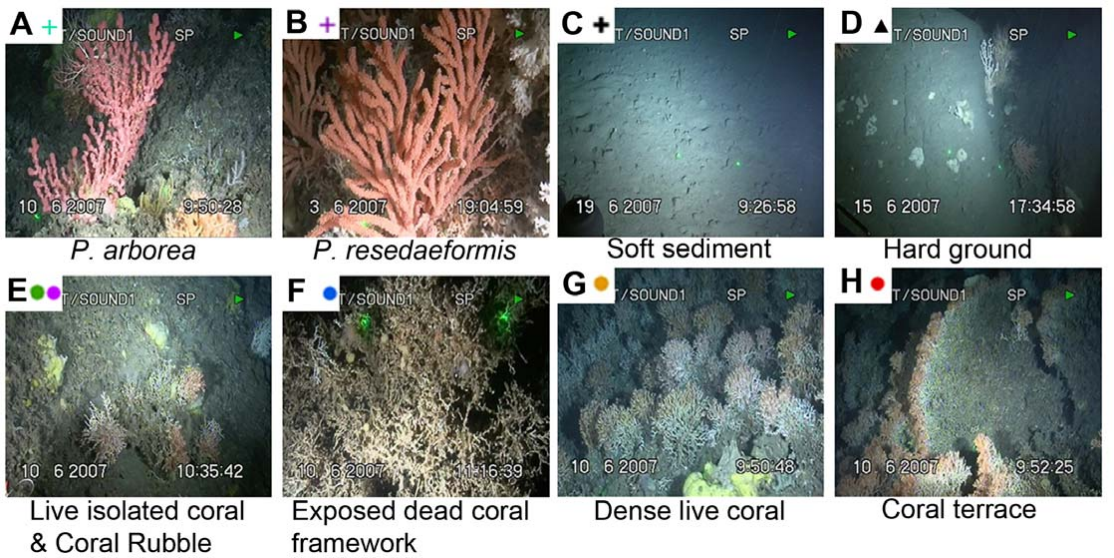
Applied categories of substrate presence.

Røst Reef, Traena Reef and Sotbakken Reef are three reef complexes located on the Norwegian margin, at depths of 250–400 m (Figure 1), with living DWCs present (Figure 2). The Sotbakken Reef and Røst Reef have been reviewed as part of the Norwegian seabed mapping program MAREANO [28], [29], [30]. The water mass at the three reef complexes is of Atlantic origin [31], with a temperature >5°C and salinity >35‰ [32].

The Røst Reef was discovered off the SW tip of the Lofoten islands by the Institute of Marine Research (IMR), Norway in May 2002 [33]. The reef complex is 35–40 km long and up to 3 km wide, situated along a rugged part of the shelf break characterized by steep seabed gradients [33].

The Sotbakken Reef is located on a triangular plateau on the upper part of a large submarine slide (∼30 km in width) situated in the cross-shelf trough Håkjerringdjupet off Tromsø between Fugløybanken and Nordvestbanken [34].

The Traena Reef complex is located in a sheltered embayment on the northern edge of the cross-shelf trough Traenajupet [35]. The complex consists of a field of reefs of similar size elongated in a similar direction, suggesting that reef development may be directly related to the local hydrodynamic conditions.

### Data Acquisition and Analysis

The majority of the data used in this study was acquired during the Polarstern ARK XXII/1a expedition (2007) [36], comprising of ship-borne multibeam echosounder bathymetry data at Røst Reef and Sotbakken Reef, time-stamped videos from nine JAGO (IFM-GEOMAR) submersible dives and associated JAGO dive positioning data. Ship-borne multibeam bathymetry data collected from Traena Reef by IMR was also used in this study [35].

The multibeam bathymetry data was logged by a Hydrosweep DS-3 multibeam sonar data acquisition system at Røst Reef and Sotbakken Reef and further processed using the open source software MB-system. The processed data was projected from longitude and latitude on the WGS84 reference ellipsoid to UTM Zone 32N coordinates at the Røst Reef, and to UTM Zone 34N coordinates at the Sotbakken Reef, and gridded to 10 m resolution raster. The bathymetry data at Traena Reef was provided in fully processed format with 10 m resolution and WGS 84 UTM Zone 32N coordinates. The positioning data for the JAGO dives was cleaned of erroneous readings by removing outliers, assuming a maximum JAGO survey speed of ∼0.8 m s^−1^. The data was further cleaned using a Gaussian smoothing technique (used software package Adelie-GIS 1.8) to decrease the random error in the positioning data.

The presence of *P. arborea* and *P. resedaeformis* colonies was logged using the software package OFOP 3.2.0j (Ocean Floor Observation Protocol) throughout each dive video, with locations determined by comparison with the associated, cleaned and timecode-stamped JAGO submersible positioning data. In addition, the substrate was characterized throughout each dive video, with the following categories used: coral rubble, exposed dead coral framework, live isolated coral framework, dense live coral, coral terrace, soft sediment, hardground, pebbles/cobbles, boulders (Figure 2). Throughout the video transects, the predominant (greatest frame coverage) of these substrate categories present within the video was logged. These categories followed those used in a published study of the Sula Reef complex on the Norwegian margin [37]. Regions of seafloor were defined as ‘Coral terrace’ in the current study if they consisted of large *L. pertusa* frameworks, either exhibiting the ‘inclined wall’ growth morphology with live polyps on crest (see Figure 2H), as often observed at Røst Reef, or exhibiting the ‘cauliflower’ morphology with live polyps on the framework surface and radiating outwards from its center, as often found at Sotbakken Reef.

### Multiscale Terrain Variables

In this study, 20 multiscale terrain variables (seabed topography descriptors) were calculated at scales of 30 m, 90 m and 170 m, corresponding to moving window sizes of 3×3, 9×9 and 17×17 cells, from the gridded multibeam bathymetry data for each of the study sites (Table 1). The scales used were adopted based on the assumption that terrain variables at the selected spatial scales may correlate with the distribution of the studied species [12], [18], [21], [38].

**Table 1:** Multiscale terrain variables applied in this study.

Terrain variable	Moving window size (m)			Software
	30×30	90×90	170×170	
Slope	SLO3	SLO9	SLO17	Landserf 2.3
Aspect	ASP3	ASP9	ASP17	Landserf 2.3
Mean Curvature	MEC3	MEC9	MEC17	Landserf 2.3
Plan Curvature	PLC3	PLC9	PLC17	Landserf 2.3
Profile Curvature	PRC3	PRC9	PRC17	Landserf 2.3
BPI	BPI3	BPI9	BPI17	ArcGIS 9.2
Rugosity	RUG3			ArcView 3x. ext.
TRI	TRI3			ArcGIS 9.2 macro

In the current study, slope has relevance in that it influences current flow amplification [12], [21], with consequences for the delivery of food particles to the corals. Aspect provides information on orientation of a region of the seabed, and therefore the degree to which the seabed terrain is exposed to local and regional prevalent currents. Aspect may therefore be important in determining benthic habitat morphology and community structure, and is likely of high importance for suspension-feeding fauna [12] such as the gorgonians in this study. DWCs are prevalent on topographic highs and therefore colony concentrations may be associated with the relative position of a region of seabed in relation to the surrounding area. Here, the frequently used descriptors of seabed relative positions – BPI, plan curvature, profile curvature and mean curvature [17], [21], were derived at the selected multiple scales to investigate their relative importance on the distribution of *P. arborea* and *P. resedaeformis*. Certain species (such as *P. arborea* and *P. resedaeformis*) are most numerous in complex habitats with strong structural components (e.g., on rocky outcrops), whereas other species tend to occupy soft sediment on flat terrain [21]. Therefore, change in seabed terrain complexity is often associated with changes in species colonization. For this investigation, two frequently used descriptors for terrain complexity – rugosity and terrain ruggedness index (TRI, a measure of the local depth variation compared to the focal cell of the analysis window [12], [17], [18], [21]), have been applied to test their relative importance on the distribution of *P. arborea* and *P. resedaeformis*.

### Multivariate Statistics Method – Ecological Niche Factor Analysis

Ecological Niche Factor Analysis (ENFA) is a multivariate statistical method, which transforms a number of possibly correlated variables into the same number of uncorrelated factors. But unlike the similar method of Principal Component Analysis, the factors have ecological meaning, with the first factor accounting for the marginality information and part of the specialization information of species distribution, and the following factors accounting for the remaining specialization information [39], [40]. Marginality represents the degree to which the species realized ecological niche differs from the mean available habitat, therefore aiding in determining the preference of the species for specific environmental conditions within the available space. Specialization is defined as the ratio of the ecological variance of the mean available habitat to that observed for the focal species, therefore quantifying the narrowness of the species realized ecological niche with regard to particular environmental variables [40].

In this study, the terrain variables were first normalized by applying the Box-Cox algorithm [40]. ENFA (used software package Biomapper version 4.0) was performed on *P. arborea* at Sotbakken Reef by using the 20 terrain variables outlined in Table 1. For both species at Røst Reef and Traena Reef, depth was added as a further terrain variable. The marginality (normalized) and specialization of species distribution contributed by each terrain variable were then calculated using Biomapper. The specialization values were manually normalized. The contribution of each terrain variable was calculated as the mean value of the marginality value and normalized specialization value contributed by the corresponding variable, and further manually normalized. The higher the marginality value, the further the species distribution departs from the mean available habitat for that terrain variable [17], [18], [21]. The higher the specialization value, the more narrowly focused the species distribution is with regard to the corresponding variable [40]. The higher the normalized contribution value, the more closely related the terrain variable is with the species distribution. The important terrain variables were identified by comparing their corresponding normalized contribution values. The species habitat selection was assessed by analyzing the coefficients of the marginality factor (with a positive coefficient indicating preference for higher-than-mean values for that terrain variable, and vice versa) and the normalized specialization contributed by each terrain variable.

## Results

In total, 754 gorgonian colonies were recorded from nine video transects, over a distance of 3.8 km, with 51.7% and 73.1% occurrences of *P. arborea* and *P. resedaeformis* colonies, respectively, observed at the Røst Reef (Table 2). Additional results were analyzed for each coral reef complex.

**Table 2:** *P. arborea* and *P. resedaeformis* occurrence observed at three study sites by JAGO dives.

Transect	At reef complex	Length (m)	Observed colonies	
			*P. arborea*	*P. resedaeformis*
Dive1	Røst Reef	485	27	38
Dive2	Røst Reef	489	104	104
Dive3	Røst Reef	406	65	132
Dive4	Sotbakken Reef	542	67	10
Dive5	Sotbakken Reef	674	51	8
Dive6	Sotbakken Reef	332	7	0
Dive7	Traena Reef	312	30	36
Dive8	Traena Reef	365	23	43
Dive9	Traena Reef	197	5	4
sum		3802	379	375

The length given in the table is the sum of length sections of JAGO dive transect with usable video.

### Røst Reef

**Figure 3:**
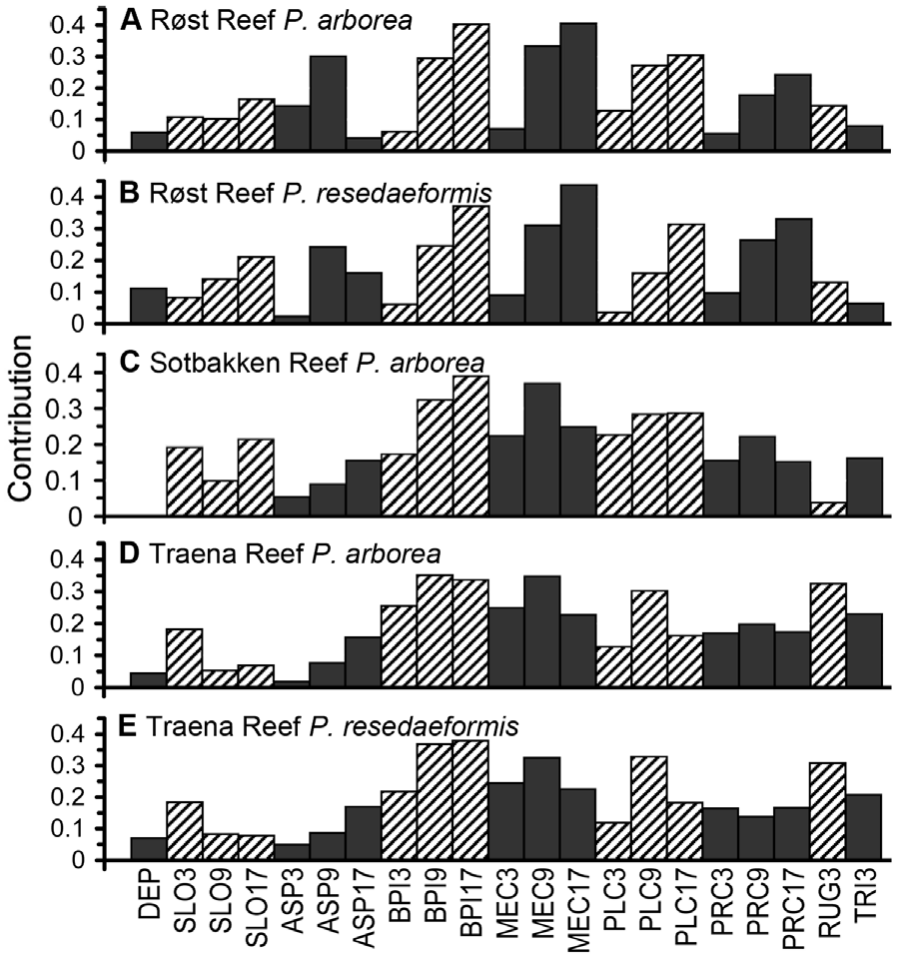
Contribution of each terrain variable to observed distribution of species.

**Figure 4:**
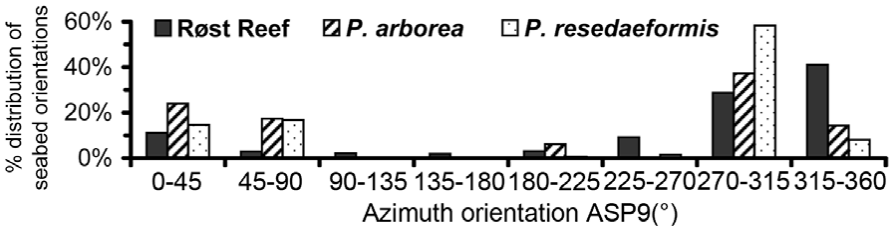
Percentage distribution of ASP9 of the overall available habitat, *P. arborea* observed localities and *P. resedaeformis* observed localities at Røst Reef.

Figure 3A and 3B show that BPI and curvature at analysis scales of 90 m and 170 m were most closely correlated with the observed distribution of both *P. arborea* and *P. resedaeformis* at Røst Reef, with the correlation strength increasing with the analysis scale increases. Additionally, seabed aspect (ASP9) was found to be closely related with the distribution of both species (particularly *P. arborea*) at Røst Reef (Figure 3A, 3B). Figure 4 indicates that both species were observed with a relatively greater abundance in areas with seabed orientation of [45°, 90°] on variable ASP9. The high abundance of *P. arborea* was also observed in association with seabed aspect orientation of [0°, 45°]. For *P. resedaeformis*, high abundance was also observed in areas with [270°, 315°] azimuth orientation.

**Figure 5:**
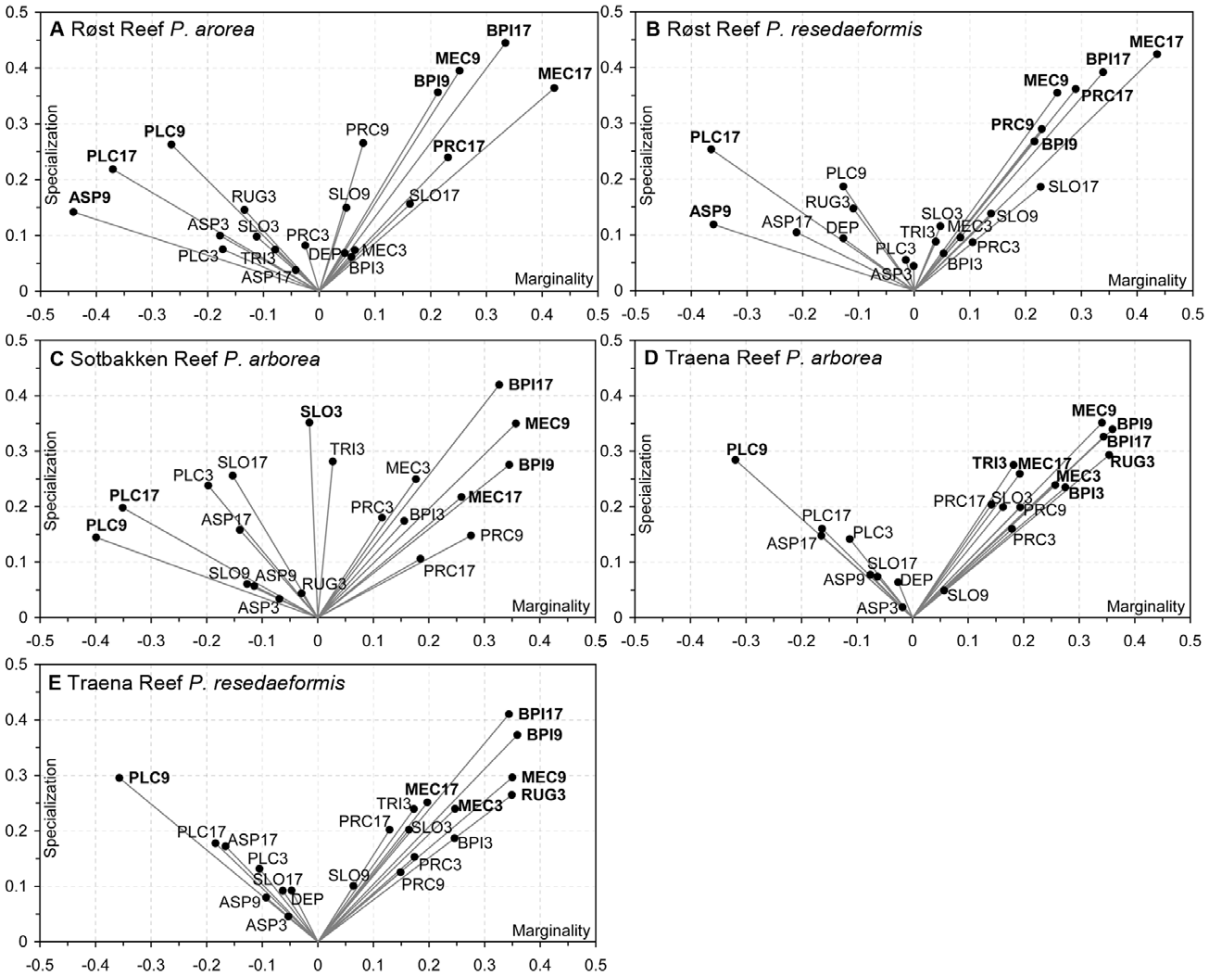
Biplots of the marginality and specialization of species distribution contributed by each terrain variable. Those terrain variables making the largest contribution (the absolute of x or y>0.3, or the sum of absolute x and y>0.45) are highlighted in bold.

The positive marginality coefficients of BPI, mean curvature and profile curvature, and the negative marginality coefficients of plan curvature (Figure 5A, 5B) indicated both species tended to inhabit topographic highs with higher values of BPI, mean curvature and profile curvature, and lower values of plan curvature than mean available habitat at Røst Reef. Terrain variables PLC9, ASP9 and PRC9 were found to contribute differently to the marginality of the observed distribution of *P. arborea* and *P. resedaeformis*. PLC9 and ASP9 contributed to the marginality of *P. arborea* distribution much more than to the marginality of *P. resedaeformis* distribution, whilst PRC9 contributed to the marginality of *P. resedaeformis* distribution to a much greater extent than to marginality of *P. arborea* distribution.

**Figure 6:**
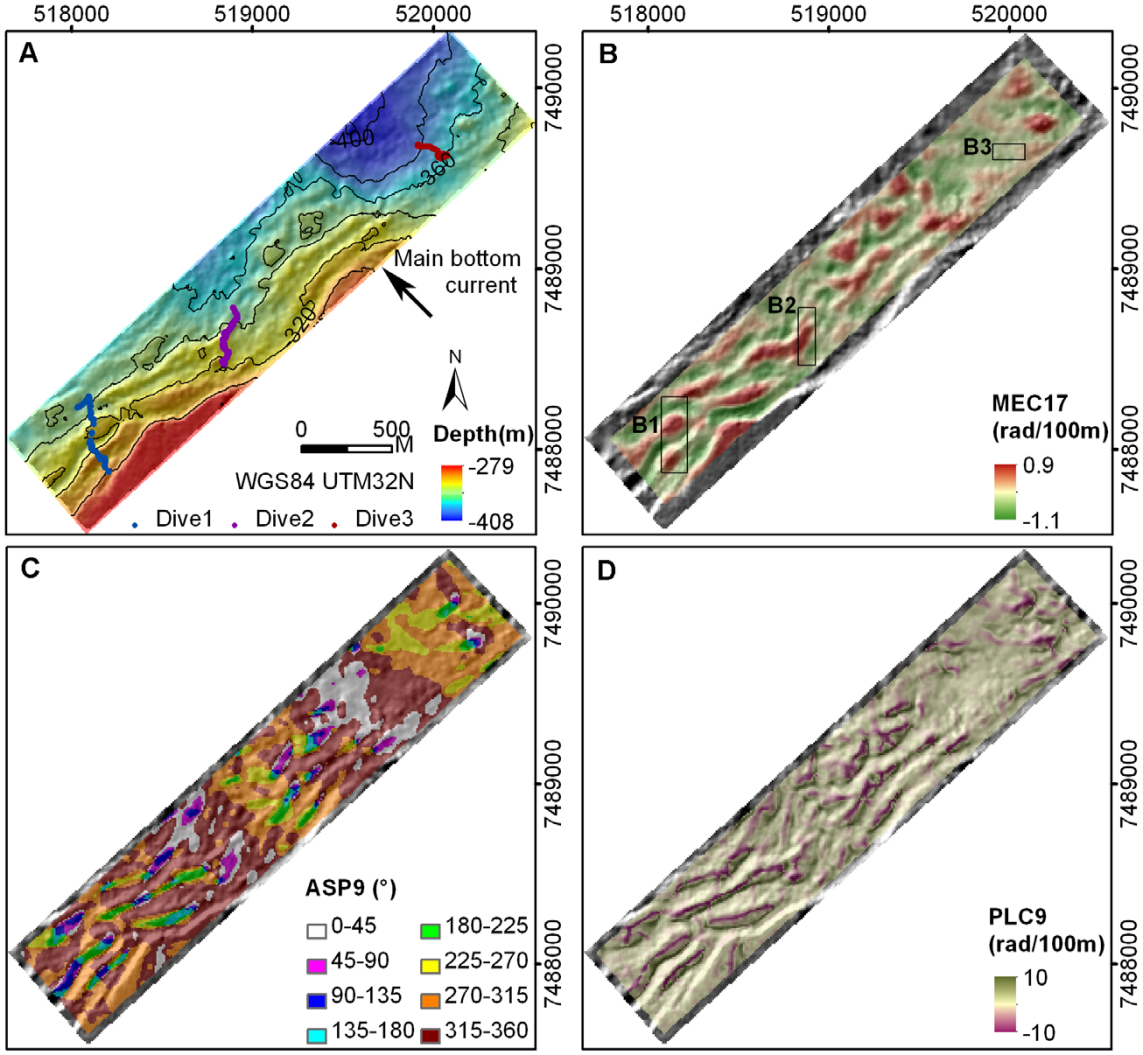
Bathymetry and terrain variables at Røst Reef. (A) bathymetry with three JAGO dive transects and main bottom current direction, (B) MEC17, (C) ASP9, (D) PLC9.

**Figure 7:**
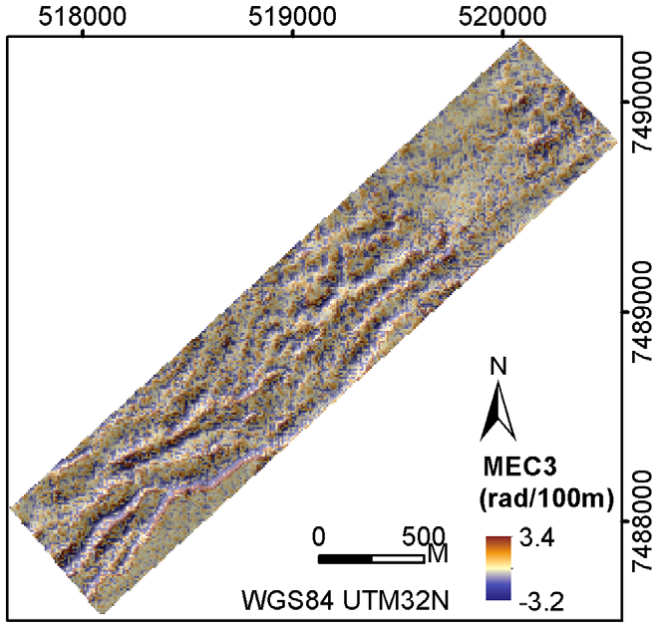
Terrain variable MEC3 at Røst Reef.

Bathymetry data showed the surveyed region of the Røst Reef to be characterized by steep ridges of up to ∼150 m width, running in a SW–NE direction on the shelf break, with the shelf sloping in a north westerly direction (Figure 6A). Parameter MEC17 highlighted the crests of the large ridges (those with widths of approx. >120 m). Where a larger ridge was closely spaced with small neighboring ridges, MEC17 commonly smoothed and captured these ridges as one large ridge-like feature, highlighting the crest of the ridge-like feature in red (Figure 6B). ASP9 illustrated the ridges as a spatially scalariform array facing toward the NW (Figure 6C). PLC9 captured the uppermost crests of the ridges as topographic divergences (pink) and the seabed depression areas between the ridges as the topographic convergences (green) (Figure 6D). The smaller-scale parameter MEC3 was effective at capturing the detailed terrain variations and highlighting the local topographic reliefs (dark yellow) (Figure 7).

**Figure 8:**
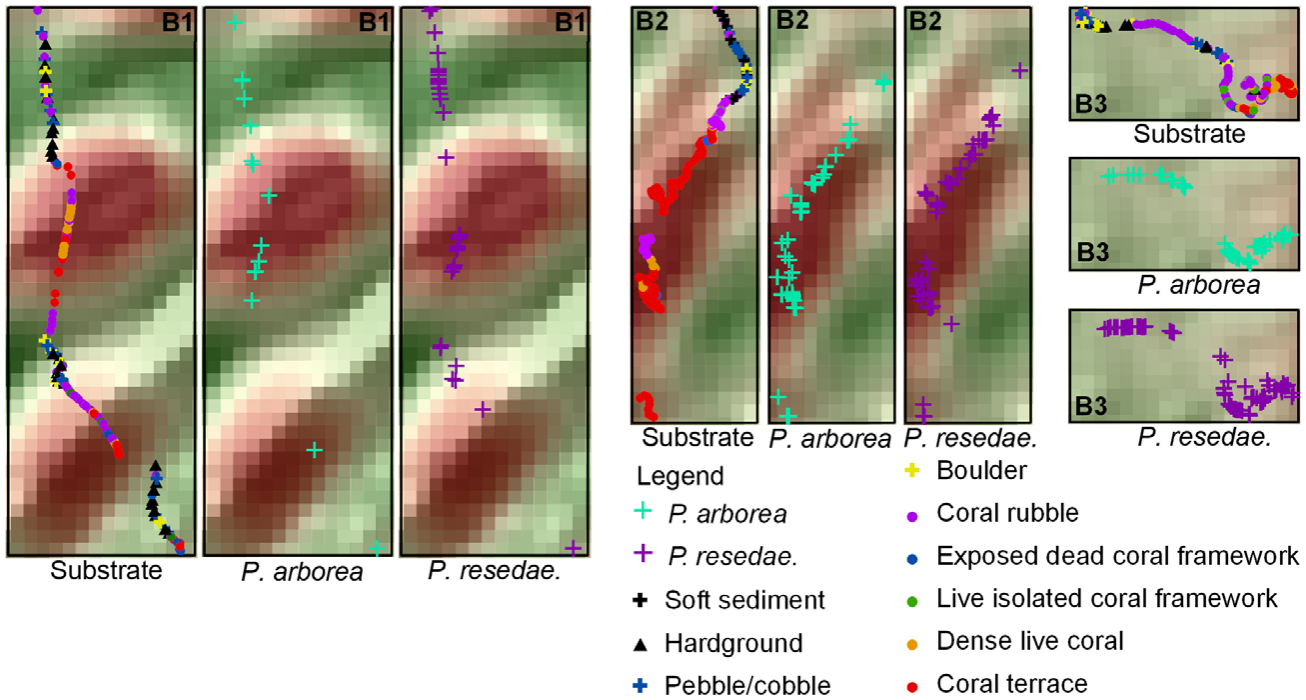
Detailed plots of boxed areas B1, B2 and B3 (from Figure 6B). The substrate presence, *P. arborea* and *P. resedaeformis* occurrence along the JAGO dive transects were shown for each corresponding area.

At Røst Reef, two JAGO dive transects (Dive1, Dive2) traversed the ridges, with the third (Dive3) surveying the upper slide (Figure 6A). Dense live *L. pertusa* colonies and *L. pertusa* terraces were observed mainly on the upper section of the ridges (particularly on the east facing side) (Dive1, Dive2), and of the relief structure situated on the upper slide (Dive3) (Figure 8). The *L. pertusa* terraces with live polyps on surface, predominantly faced in east to south direction. Substrate categories of hardground, pebbles/cobbles, and boulders were often observed on lower sections of the ridges and in seabed depressions between ridge structures. Both gorgonian species were observed abundantly distributed on coral framework or rubble on the ridges. A percentage of both species were also found on hard substrates and on boulders/cobbles on lower section of the ridges and in the seabed depressions (Figure 8).

### Sotbakken Reef

Figure 3C indicates that BPI and curvature (BPI and plan curvature at scales of 90 m and 170 m and MEC9) were most closely correlated with the observed distribution of *P. arborea* at Sotbakken Reef. *P. arborea* tended to inhabit positions with higher values of BPI, mean curvature, profile curvature, and lower values of plan curvature than mean available habitat, indicated by the coefficients of the marginality factor (Figure 5C).

**Figure 9:**
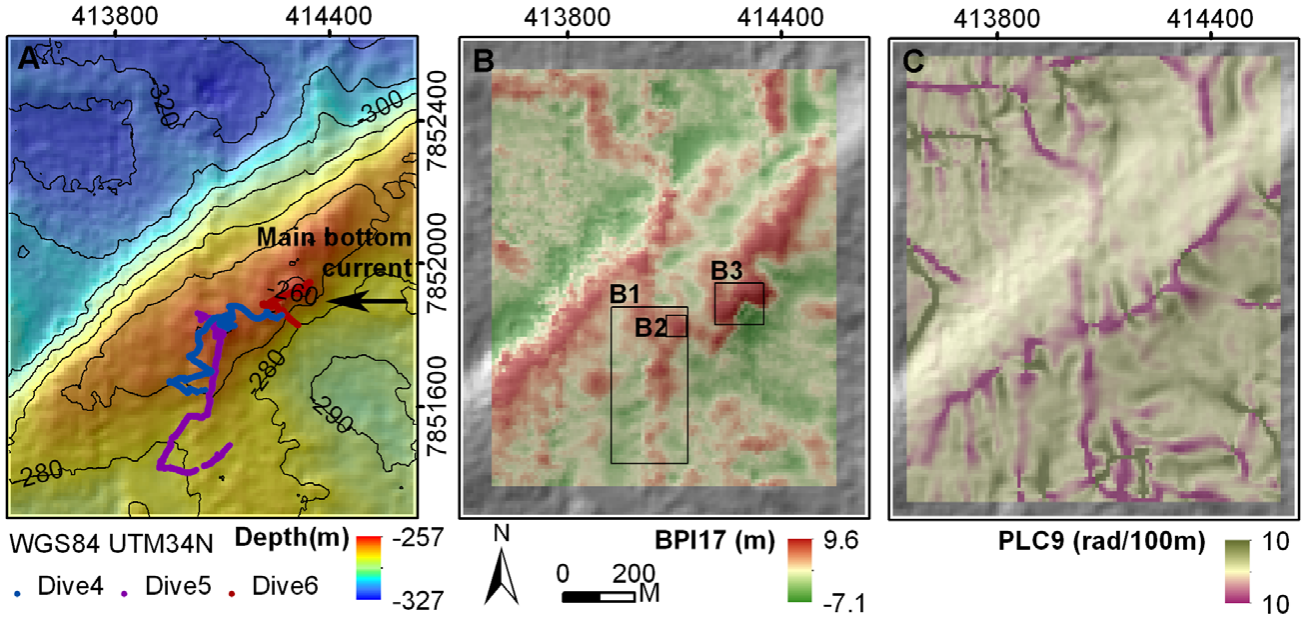
Bathymetry and terrain variables at Sotbakken Reef. (A) bathymetry with three JAGO dive transects and main bottom current direction, (B) BPI17, (C) PLC9.

Bathymetry data revealed that the seabed topography at Sotbakken Reef is characterized by a large plateau in the SE area, with a slope value of ∼14° (SLO17) to the NW (Figure 9A). A large mound is located on the NW extremity of this plateau, ∼20 m above the surrounding plateau and extending more than 1 km in a SW–NE direction. BPI17 highlighted the upper steep slope (red), and sections on crest of the mound (red) such as the high relief structures (∼100 m diameter) (Figure 9B). PLC9 captured the topographic divergence area elongated in a SW–NE direction on crest of the large mound, and highlighted the detailed sections of local topographic highs in pink and local depressions in-between in green (Figure 9C).

**Figure 10:**
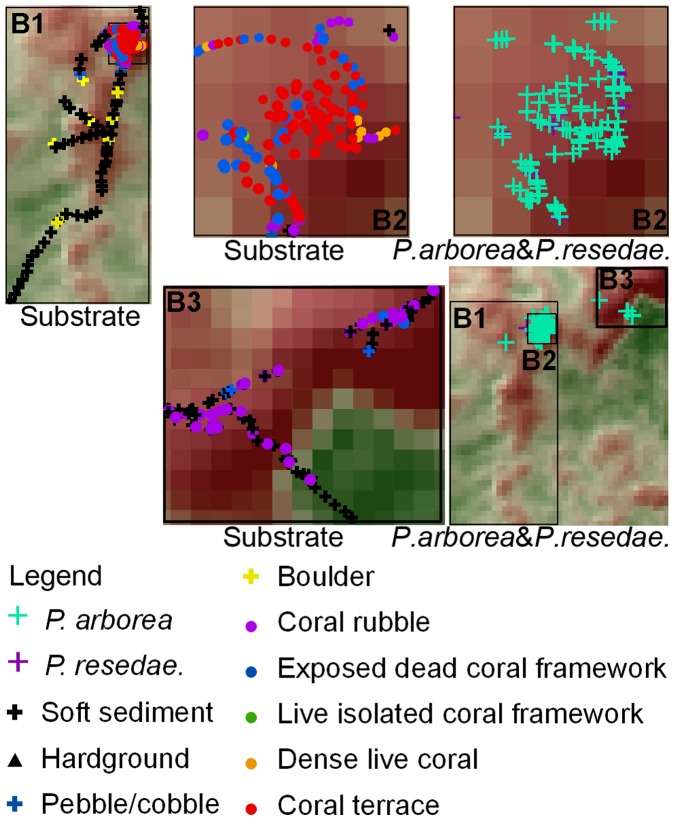
Detailed plots of boxed areas B1, B2 and B3 (from Figure 9B). The substrate presence, *P. arborea* and *P. resedaeformis* occurrence along the JAGO dive transects were shown for each corresponding area.

At Sotbakken Reef, three submersible transects crossed the crest of the mound and its SE flank (Figure 9A). Substrate type observed across this flank was predominantly soft sediment with some boulders or pebbles/cobbles (Figure 10). Coral rubble and soft sediment with some pebbles/cobbles were observed across the eastern crest of the mound. Large *L. pertusa* frameworks were observed throughout the high relief structure on the western crest (Figure 10), with colonies exhibiting the ‘cauliflower’ growth morphology [41] and forming terraces around the relief structure and outwards from its center. Additionally, both gorgonian species were found concentrated on the *L. pertusa* reef on the western crest. The large fan-shaped *P. arborea* colonies were often observed with the concave side facing in a NE to SE direction.

### Traena Reef

Figures 3D and 3E indicate that BPI and curvature (BPI9, BPI17, MEC9 and PLC9) were the variables which most closely correlated with the observed distribution of *P. arborea* and *P. resedaeformis* at Traena Reef. Additionally, terrain variability RUG3 was also relevant to distribution of both species, and TRI3 also relevant to *P. arborea* distribution at this site. Both species were most prevalent in positions with higher values of BPI, mean curvature, profile curvature, RUG3, TRI3 and lower values of plan curvature than mean available habitat, as indicated by the coefficients of the marginality factor (Figure 5D, 5E).

**Figure 11:**
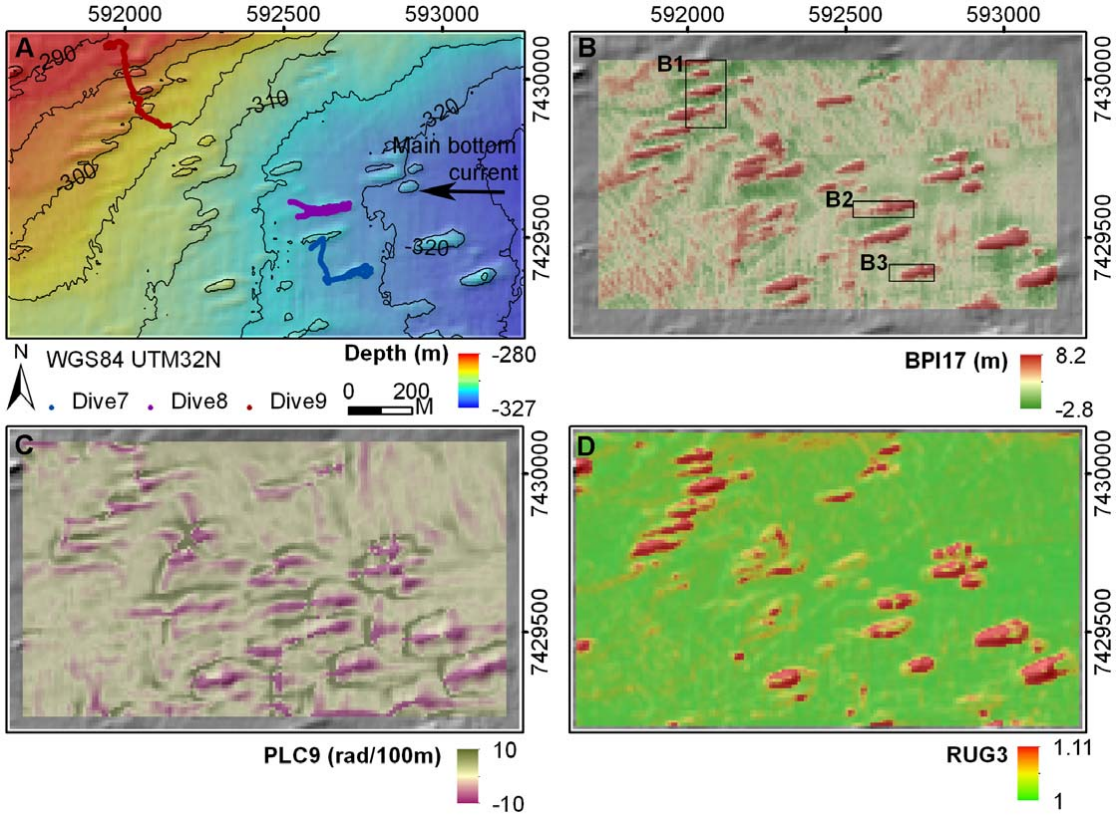
Bathymetry and terrain variables at Traena Reef. (A) bathymetry with three JAGO dive transects and main bottom current direction, (B) BPI17, (C) PLC9, (D) RUG3.

The bathymetry data collected by IMR showed the Traena Reef complex to be characterized by many reefs elongated in a WSW–ENE direction, each with approximate dimensions of 30–60 m width, 100–180 m length and 5 m height (Figure 11A). BPI17 highlighted the upper sections of these reef structures in red (Figure 11B). PLC9 differentiated the crests of reef structures as divergent areas of the seabed (pink) surrounded by convergent areas (green) (Figure 11C). RUG3 effectively captured the relative flat reef crests and high terrain complexity of the reef slopes (Figure 11D).

**Figure 12:**
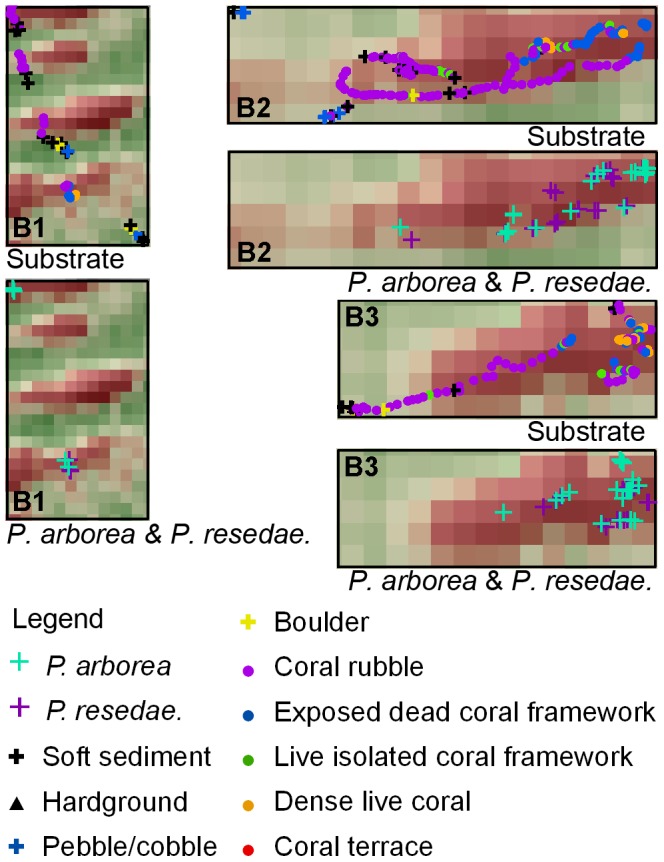
Detailed plots of boxed areas B1, B2 and B3 (from Figure 11B). The substrate presence, *P. arborea* and *P. resedaeformis* occurrence along the JAGO dive transects were shown for each corresponding area.

At Traena Reef, the three submersible transects crossed the crests and sides of a number of the elongated reefs (Figure 11A). Dense coral framework (mostly dead) was observed on the eastern tip of the reefs, with dense coral rubble logged throughout the western region of the reefs (Figure 12). Fine-grained sediment (silt to clay and biogenic debris) with some cobbles and boulders was observed on the seafloor between the reefs. Highest densities of both gorgonian species were observed at the eastern section of the reefs, usually on coral framework or rubble substrate.

## Discussion

### Key Terrain Variables Across Study Sites

The terrain variables examined in this study had similar correlation patterns with distribution of *P. arborea* and *P. resedaeformis* at each particular study site of Røst Reef and Traena Reef, but different correlation patterns with species distribution across different study sites (Figure 3). This would suggest that the terrain variables closely correlated with the distribution of these two species appear to differ across study sites with different geographical settings, but are similar at a particular reef complex. In this study, the variables slope, aspect, RUG3 and TRI3 varied in ecological relevance to distribution of these two species across the study sites, whilst the variables BPI and curvature, particularly BPI, mean curvature and plan curvature at analysis scales of 90 m or 170 m, proved to be most relevant to the observed distribution of *P. arborea* and *P. resedaeformis* at Røst Reef and Traena Reef, and to *P. arborea* distribution at Sotbakken Reef (Figure 3).

The terrain variables of BPI and curvature have been reported to be strongly correlated with *L.pertusa* coverage [12], and are considered important in habitat suitability modeling for distribution of DWCs such as *Madrepora oculata* and *L. pertusa* [17], *L. pertusa* isolated colonies and/or *L. pertusa* reefs [18], [25] at analysis scales of meters to hundreds of meters. The importance of BPI and curvature in habitat predictions highlights the importance of the relative position of focal localities to the surrounding terrain in influencing distribution of the investigated DWCs. Given that the three reef complexes investigated in this study varied greatly in morphology and underlying topography, the distribution of *P. arborea* and *P. resedaeformis* was most closely correlated with the topographic properties BPI and curvature. We hypothesize that at other comparable study sites (such as other locations with similar depth and substrate availability on the European margin), similar close relationship may also exist. Additionally, the degree to which the distribution of *P. arborea* and *P. resedaeformis* correlated with terrain variables is scale-dependent. The selection of suitable analysis scales of terrain variables is therefore important in habitat prediction, to ensure features which most influence distribution of a target species are captured.

### Scale-dependent Terrain Variables Correlated with Coral Distribution

The distribution of *P. arborea* and *P. resedaeformis* in relation to terrain variables is scale-dependent. At Røst Reef, the BPI, mean curvature, plan curvature and profile curvature at analysis scales of 90 m and 170 m, and ASP9 proved to be most relevant to the observed distribution of both species (Figure 3A, 3B). This result suggests the importance of the ridge structures captured by these variables at a scale of 90 m, and the large ridges or ridge-like features captured by the variables at a scale of 170 m, in influencing both species’ distribution (Figure 6). At Sotbakken Reef, BPI and plan curvature at scales of 90 m and 170 m, and MEC9 were found most closely related to *P. arborea* distribution (Figure 3C), with these variables highlighting particular features of the large mound such as the high relief structures on the crest (Figure 9). At Traena Reef, the BPI9, BPI17, MEC9, PLC9 and RUG3 proved to be most closely related to distribution of both species (Figure 3D, 3E), with these variables capturing the individual reef structures (Figure 11).

At continental margins, DWCs rely on the delivery of phytodetritus, zooplankton, or particulate organic matter derived from near-surface primary production [42], [43], [44], [45], [46], [47], [48], [49], often via lateral advection within the benthic boundary layer [44], [50]. In order to maximize encounter rates with food particles, DWCs adjust their colony growth morphologies to face into the prevailing bottom current [1], [2], [7]. At Røst Reef, the *L. pertusa* structural terraces with dense live polyp coverage were observed to be oriented predominantly SE, as were the concave side of the observed *P. arborea* colony fans. Such colony growth morphologies would indicate that the food material was likely transported prevalently in a NW direction via cross-shelf transport [7], [15], [19]. Two short duration ADCP deployments (18 and 24 hours) at Røst Reef taken during the same research cruise also indicated bottom current flow in a NW direction [36]. At Sotbakken Reef, the concave surface of the large *P. arborea* colonies was predominantly observed facing between NE and SE, indicating the prevalent bottom current as likely flowing in a westerly direction. At Traena Reef, dense dead and some living coral framework were observed in the eastern area of the parallel reefs elongated in WSW–ENE direction (Figure 12). Such a growth morphology indicates a westerly flowing prevalent bottom current [3], again supported by a 24-hour ADCP deployment at Traena Reef carried out during the same cruise [36].

Local topography can strongly influence bottom current velocity [51], [52], [53]. The terrain features such as ridges or ridge-like features, captured by these important terrain variables at Røst Reef, may result in acceleration of the bottom current velocity (cross-shelf, prevalently NW flowing) on the upstream side of the ridge structures or ridge-like features [19]. Enhanced bottom currents have been shown to be important for food supply to DWCs [52], [54], [55], [56], increasing the transport of particles in benthic boundary layer either down towards the seabed or in from the surroundings to where DWCs can catch them [19]. Additionally, high bottom current velocity prevents living coral colonies from being buried by sediment, ensuring periodic clearance of the coral surface of deposited material [51], [53]. The mound/reef topography induces localized increasing current velocity and mixing, which may promote the advection of food particles for utilization by suspension feeders (such as DWCs) in some regions of the mound/reef structure [46], [52], [56], [57]. Locations such as the relief structures on the crest of the mound highlighted by the most important terrain variables at Sotbakken Reef and the upper sections of the reef structures highlighted at Traena Reef are examples of those likely to benefit from this increased food flux.

The correlation between variables BPI and curvature and the observed distribution of both species increased with the analysis scale increases at Røst Reef, indicating the possibility of even stronger correlation at larger analysis scales. This suggests the potential importance of even larger scale terrain features at the Røst Reef, in influencing distribution of both species, those at a larger scale than investigated here. A tidal current regime has been reported at *L. pertusa* reefs on the Norwegian shelf [58], [59]. It is possible that large ridge-like features of hundreds of meters diameter may have a pronounced impact on residual and tidal flow, such as reported at the Galway Mound on the Irish Margin [56], thus influence coral distribution. Traena Reef will not be exposed to such a tidal effect as these large scale features are absent at the location. This absence is likely the reason why the increasing contribution of BPI and curvature observed at Røst Reef with increasing analysis scale is not also observed at Traena Reef. This hypothesis is supported by Mortensen & Buhl-Mortensen [1], who report the distribution and abundance of gorgonian corals to be positively related to large-scale topographic features such as shelf breaks and ridges, as qualitatively assessed from bathymetric map data in the Northeast Channel at Atlantic Canada.

In this study, the terrain variables of BPI and curvature at the 30 m analysis scale were shown to have a lesser relevance than those at 90 m or 170 m analysis scales to the observed distribution of *P. arborea* and *P. resedaeformis*, particularly at Røst Reef. These variables at a scale of 30 m captured detailed terrain variation, rather than the larger features such as the ridge structures at Røst Reef, or the topographic relief structures on the crest of the mound at Sotbakken Reef, or the reef structures at Traena Reef. Additionally, bathymetric noise may account for some of the observed variability to a greater degree at this scale than at larger scales [38]. The resolution of ship-borne bathymetry data used in this study is not high enough to calculate the terrain variables at a scale relevant to the variation of macro habitat (1–10 m).

### Assessing Terrain Habitat Selection

At each of the three geographically distinct study sites, *P. arborea* and *P. resedaeformis* tended to inhabit positions with higher values of BPI, mean curvature and profile curvature, and lower plan curvature (at 90 m or 170 m analysis scale) than the mean available habitat, shown by ENFA (Figure 5). This indicates that both species have a tendency to occupy topographic highs with slope increasing downhill (typically on upper slopes) or topographic divergence, such as the upper sections of the ridges or the large ridge-like features at Røst Reef, the upper flanks of the large mound and relief structures on the crest of the mound at Sotbakken Reef, and the upper sections of the reef structures at Traena Reef. Such habitat preferences have been reported for DWCs elsewhere [1], [3], [5], [12], [13], [17]. Such topographic highs are normally associated with hard substrates, and strong bottom current velocities [5], [15], [16], [60], indicating likely elevated food availability for benthic species.

Both *P. arborea* and *P. resedaeformis* were observed in high abundance on structural *L. pertusa* frameworks at the three study sites. Examples of such structures include the dense frameworks located on the upper sections of the ridge structures or the ridge-like features at Røst Reef (Figure 8), the flourishing *L. pertusa* reef on the western crest of the mound at Sotbakken Reef (Figure 10), and the coral frameworks at the eastern tips of the reefs at Traena Reef (Figure 12). The physical structure of *L. pertusa* coral framework increases local turbulence, thereby increasing inflowing food flux and making a greater volume of material available for DWCs [45]. Additionally, such dense coral frameworks may act as a trap for particles, including primary pelagic, resuspended and locally produced benthic material released by fauna [54], [61]. The resuspension of freshly deposited particles during periods of heightened flow velocity will often make available to corals the same material a number of times, possibly regularly in tidally driven locations [54]. At the time of the reef surveys, the bottom current velocities exceeded 15 cm s^–1^ (the critical speed required for resuspension of surface sediment in the NE Atlantic continental margin [62]) for a percentage of the time that the ADCP flow meters were deployed [36], indicated that periodic resuspension of settled material is likely a recurring feature at Røst Reef and Traena Reef.

A percentage of both *P. arborea* and *P. resedaeformis* colonies were observed in topographic low-lying areas with negative MEC17 values, e.g. the lower sections of the large ridge structures or ridge-like features and the depressions in-between these structures at Røst Reef (Figure 8). The substrate within these areas was dominated by hardground, pebbles/cobbles or boulders, which are likely to aid the gorgonian corals in settlement. At Traena Reef and Sotbakken Reef, almost all colonies were observed at topographic highs with positive BPI17 (Figure 10, 12). The widely distributed soft sediment around the reefs at Traena Reef complex, and the soft sediment around the relief structures on the western crest of the mound at Sotbakken Reef, may be an additional factor limiting the spreading of corals locally (Figure 10, 12). The remaining coral rubble on the eastern crest of this mound with few colonies of each gorgonian species observed at Sotbakken Reef is likely a result of heavy fishing activities in this area.

A difference in distribution of *P. arborea* and *P. resedaeformis* at Røst Reef was indicated by the different contribution of PLC9/PRC9/ASP9 to the marginality of each species distribution (Figure 5A, 5B). *P. arborea* tended to inhabit the topographic divergences such as on the crest of ridges to a greater degree than did *P. resedaeformis*, whilst *P. resedaeformis* tended to inhabit areas with slope increasing downhill such as on the upper slopes of ridge features stronger than *P. arborea*. At Røst Reef, a higher abundance of *P. arborea* was observed on the crest and east-facing side of the large ridges (ASP9 [0°, 45°] and [45°, 90°]), whilst *P. resedaeformis* was abundant on the east-facing side of the ridges and the small relief structures on the relatively smooth upper slide with west-facing direction (ASP9 [45°, 90°] and [270°, 315°]) (Figure 4). The two species are filter feeding organisms, capturing food passively from that maintained in suspension by benthic currents. The fan-shape structures of *P. arborea* colonies have been hypothesized to increase food capture efficiency under flow [5], whilst *P. resedaeformis*, with an irregular bushy morphology, appears to utilize a more refractory, resuspended food supply than the more erect *P. arborea* [2]. The potential difference in diet and/or food collection strategy between the two species may partly explain the slight difference in the distribution of *P. arborea* and *P. resedaeformis*. However, such a difference in the tendency of the distribution of the two species was not clear at Traena Reef.

### Conclusions

This study investigated the relationship between the distribution of *P. arborea* and *P. resedaeformis* and terrain variables at locations on the Norwegian continental shelf using the multivariate statistics approach ENFA. A similar correlation between species distribution and terrain variables was found at each particular study site of Røst Reef and Traena Reef. The variables of curvature and BPI at analysis scales of 90 m or 170 m, particularly BPI, mean curvature and plan curvature, were most closely related to the distribution of *P. arborea* and *P. resedaeformis* at Røst Reef and Traena Reef, and *P. arborea* at Sotbakken Reef.

Both species tended to inhabit regions of local topographic highs with positive BPI at each study site, with both species observed at Sotbakken Reef and Traena Reef almost exclusively on local topographic highs. A percentage of both gorgonian species were observed on the lower sections of the ridges or the ridge-like features, or in the bathymetric depressions between these structures at Røst Reef. In addition to the role terrain features and dense *L. pertusa* framework may play in influencing food supply, the distribution of soft sediment around the western relief structure on the crest of the mound at Sotbakken Reef and around the reefs at Traena Reef is also likely to play a role in limiting the distribution of both species locally.

Further investigation of additional environmental variables influencing coral distribution, such as the hydrodynamic conditions at both local and regional scales, would further improve our understanding of the ecological niches required by these gorgonian species.
